# Different Populations Agree on Which Moral Arguments Underlie Which Opinions

**DOI:** 10.3389/fpsyg.2021.648405

**Published:** 2021-03-15

**Authors:** Irina Vartanova, Kimmo Eriksson, Isabela Hazin, Pontus Strimling

**Affiliations:** ^1^Institute for Futures Studies, Stockholm, Sweden; ^2^School of Education, Culture and Communication, Mälardalen University, Västerås, Sweden

**Keywords:** moral arguments, moral objectivism, moral foundations, cultural universals, political attitudes

## Abstract

People often justify their moral opinions by referring to larger moral concerns (e. g., “It is *unfair* if homosexuals are not allowed to marry!” vs. “Letting homosexuals marry is against our *traditions*!”). Is there a general agreement about what concerns apply to different moral opinions? We used surveys in the United States and the United Kingdom to measure the perceived applicability of eight concerns (harm, violence, fairness, liberty, authority, ingroup, purity, and governmental overreach) to a wide range of moral opinions. Within countries, argument applicability scores were largely similar whether they were calculated among women or men, among young or old, among liberals or conservatives, or among people with or without higher education. Thus, the applicability of a given moral concern to a specific opinion can be viewed as an objective quality of the opinion, largely independent of the population in which it is measured. Finally, we used similar surveys in Israel and Brazil to establish that this independence of populations also extended to populations in different countries. However, the extent to which this holds across cultures beyond those included in the current study is still an open question.

## Introduction

Whether there is objectivity to moral claims is a long-standing controversy in philosophy. The stance that there is objective right and wrong is referred to as moral realism or moral objectivism, and it includes many nuances and subtleties (e.g., Silver, 2011; Sayre-McCord, 2017). From a practical perspective, however, the obvious lack of consensus on moral issues makes it clear that moral claims have a strongly subjective component. The case of assisted dying serves as an illustration. Some people believe a society is morally required to assist a person who wishes to die due to unbearable suffering from a terminal illness. Others believe that a moral society should severely punish anyone who gives such assistance. How can this be? Well, both sides can provide ample arguments to justify their positions (Dworkin, 2011; Keown, 2018). For example, an argument in favor of allowing assisted dying is concern about liberty, that people should have a say not only about their treatment but also about their death. An argument against allowing assisted dying is that it weakens the sacred value of human life. Thus, people may come to different judgments depending on which of these concerns they care most strongly about.

Individual differences in how strongly people care about different kinds of concerns are the focus of the moral foundations theory (Haidt and Graham, 2007; Graham et al., 2009). According to this theory, the reason that liberals and conservatives often disagree on moral questions is that only conservatives tend to assign much importance to moral arguments based on the “binding” moral foundations of authority, loyalty, and purity, whereas liberals assign especially high importance to the “individualizing” foundations of harm and fairness. This group difference has been established by large studies using the moral foundations questionnaire, the MFQ (Graham et al., 2011). Further evidence has been provided by experiments and field studies of argument exposure (e.g., Day et al., 2014; Clifford et al., 2015; Feinberg and Willer, 2015). To predict how these differences in reliance on moral concerns will guide specific moral opinions, we need a characterization of moral opinions in terms of which moral concerns apply to them. The aim of the present paper is to show how such a characterization can be obtained and demonstrate that it is largely independent of sample demographics, that is, the applicability of moral concerns is viewed similarly by women and men, by young and old, by liberals and conservatives, etc. We will even examine how the applicability of moral concerns is viewed in different countries.

## Methods for Measuring the Applicability of Specific Moral Concerns to Specific Moral Opinions

Prior research by Koleva et al. (2012) took an indirect approach. They used multiple linear regression to examine how well respondents' MFQ scores on five moral foundations predicted their moral disapproval of a given act. In this way they found that disapproval of euthanasia was “firmly linked to purity,” whereas disapproval of the death penalty “appeared to be driven not by Purity (the sanctity of life), but by Harm” (Koleva et al., 2012, p. 192). This method thus yielded plausible and interesting results. However, the indirectness of the method is a drawback. Respondents are never actually asked about what arguments they think apply to a given position. The validity of the inferred links rests on the unproven assumption that moral judgments are shaped by the individual's reliance on various moral foundations weighted by the applicability of each moral foundation to the question at hand.

More direct methods are possible. Researchers of political communication have long carried out studies where respondents are asked which arguments can plausibly be used for each position on a political issue (e.g., Cappella et al., 2002; Van der Wurff et al., 2018). These studies use open-ended questions to elicit arguments, with the aim of measuring the size of the respondent's “argument repertoire.” Thus, this research tradition focuses on the number of arguments rather than their content.

Here we will instead focus on a direct method proposed by Strimling et al. (2019). Respondents were presented with a list of general arguments adapted from the MFQ and were first asked to tick all arguments that applied to justify their own position on a given moral issue, and then tick all arguments that plausibly applied to justify the opposing position. A given moral opinion can then be characterized by an applicability score for each argument, representing how many respondents ticked the argument as applying to that opinion. Using this method in the United States, argument applicability scores for a range of moral issues were obtained by Strimling et al. (2019).

The reason for Strimling et al. (2019) to measure argument applicability was to test a theory that public opinion will move toward those moral opinions for which concerns about harm and fairness are most applicable. Thus, they examined how well opinion trends are predicted by applicability scores for arguments concerning harm and fairness. For that exercise to be meaningful, however, it is important that the applicability scores they obtained in their sample would not come out very differently in another sample. To argue for this, Strimling and colleagues used a mixed-effect model to show that individuals' political ideology and idiosyncrasies in responding accounted for only very small proportions of the total variance in their argument applicability data. Here we take a somewhat different analytical approach and ask whether argument applicability scores obtained from different subpopulations will still be essentially the same.

## Research Question

Our research question is whether argument applicability scores obtained by Strimling et al. (2019) method are objective, in the sense of being largely independent of which population is sampled. We mainly consider different subpopulations in the same country. Specifically, we consider subpopulations that prior research has linked to moral opinions: women vs. men, younger people vs. older people, liberals vs. conservatives, people with lower vs. higher education level, and people with lower vs. higher cognitive ability (e.g., Bobo and Licari, 1989; Bolzendahl and Myers, 2004). In addition, we also compare populations in a few different countries that are geographically distant to each other: the United States, the United Kingdom, Israel, and Brazil. This selection is not intended to represent the full extent of human cultures but only to provide a first test of whether views of argument applicability may be largely the same across different countries.

## A Framework for Moral Arguments

Strimling et al. (2019) used a pool of 15 moral arguments adapted from the Moral Foundations Questionnaire, where they are used to represent five different moral foundations: Harm, Fairness, Ingroup loyalty, Authority, and Purity. In moral foundations theory, each moral foundation is claimed to map to a distinct moral taste bud in humans, each with its own characteristic emotional responses and specific evolutionary history (Haidt and Joseph, 2007). For instance, Purity (i.e., ideas about taboos) is claimed to be connected with the emotion of disgust and to serve the adaptive purpose of pathogen avoidance. For the purpose of the present paper, however, these claims are irrelevant. Here we are only interested in the framework the foundations provide for categorizing moral arguments by the type of concern they voice.

The top 15 lines of Table 1 show arguments adapted from the Moral Foundations Questionnaire as well as the moral concern that each argument corresponds to according to the creators of the questionnaire (Graham et al., 2009, 2011). Strimling et al. (2019) used this list but pointed out that it is not exhaustive of arguments that can plausibly be used to support why something should or should not be allowed. For example, concerns about physical harm and violence are not covered by any of the three arguments relating to harm, which instead focus on emotional harm and suffering. This omission may be problematic when measuring which arguments apply to opinions related to violent acts, such as the death penalty. We therefore decided to include three additional moral arguments focusing on violence and physical harm. To ensure that we do not infringe on the original conceptions of moral foundations, we introduce a separate moral concern for these new arguments, which we refer to as Violence. Although it is possible that people's moral taste buds for emotional harm and violence overlap, it is not self-evident. Moreover, it makes sense to separate these moral concerns from the standpoint of argument applicability; an act may well be problematic with respect to emotional harm but not physical harm, or vice versa.

**Table 1:** The set of moral arguments used in the current study.

**Moral concern**	**Specific argument**
Harm	Someone suffers emotionally
	Someone cares for someone weak or vulnerable
	Someone is cruel
Fairness	Some people are treated differently from others
	Someone acts unfairly
	Someone is denied his or her rights
Ingroup loyalty	Someone's action shows love for his or her country
	Someone does something to betray his or her group
	Someone shows a lack of loyalty
Authority	Someone shows a lack of respect for authority
	Someone conforms to the traditions of society
	Someone creates disruption to the order in our country
Purity	Someone violates standards of purity and decency
	Someone does something disgusting
	Someone acts in a way that God would approve of
Liberty	Everyone is free to do as they wanted
	Someone's freedom of choice is restricted
	Everyone is free to decide what group norms or traditions they want to follow
Violence	Violence is used
	Someone is killed
	Someone is physically harmed
Government	It goes beyond what the government's responsibility should be
	It is expensive for the government
	The government would handle it poorly

*The first 15 arguments were used in Strimling et al. (2019)*.

Another omission in the original list is the lack of arguments covering individual liberty, that is, concerns about people's freedom being restricted. Indeed, Liberty has been proposed as a sixth moral foundation (Iyer et al., 2012). We therefore include three arguments on the theme of individual liberty. Finally, a concern often voiced in right-wing politics is what we may call Government overreach (Frankel, 2015). As the original list lacked arguments of this kind we decided to include three often voiced arguments about why the government should not involve itself: that it is not the government's responsibility, that it is expensive, and that it would handle it poorly. Note that these concerns see government as a problem and are thereby quite distinct from the concern about Authority, which entails viewing authorities and traditions as worthy of respect. In sum, to better cover the space of moral arguments that people tend to use we use a selection of 24 specific arguments corresponding to eight different moral concerns, as listed in Table 1.

## Outline of Studies

Study 1 focuses on the United States. In this study we select a large set of moral questions that have previously been asked in the General Social Survey and therefore deemed relevant for the American public. For these moral questions we employ the Strimling method to assess the applicability of moral arguments in a US sample recruited on Prolific, heterogeneous with respect to gender, age, ideology, and cognitive ability.

Study 2 focuses on the United Kingdom. We select a large set of moral questions that have previously been asked in the British Social Attitudes survey and assess the applicability of moral arguments in a British sample recruited on Prolific.

Study 3 concerns cross-national agreement between the US, the UK, Israel, and Brazil. We selected Israel and Brazil as convenient pilot cases in different continents than the US and the UK. Israel has a sufficiently large representation among users of Prolific, while we knew from previous experience that we could collect data in Brazil using facebook. In these countries we use those moral questions that overlapped between the US selection in Study 1 and the UK selection in Study 2. For these questions, which we assume to have cross-cultural relevance, we assess the applicability of moral arguments also in Israeli and Brazilian samples and then compare different countries on their argument applicability scores.

In every study we set the sample size with the aim of having each item rated by roughly 100 participants. The rationale for this number comes from the study of (Strimling et al., 2019, Supplementary Results 2), where it was found through simulations to be sufficient with 40 ratings of each item to capture the variation in argument applicability across items. As we here want to compare pairs of subsamples, such as women vs. men, we wanted each item to be rated by 40 participants in each subsample, which means at least 80 ratings per item.

## Study 1

### Method

#### Selection of Moral Questions in the United States

In the United States we use moral questions selected from the General Social Survey, abbreviated GSS, a biannual survey asking demographic, behavioral, and attitudinal questions to representative samples of American respondents (Smith et al., 2019). To be selected, an item must have “moral” content (as coded by a research assistant), see Strimling et al. (2019). Additionally, to ensure that questions have some lasting relevance, we required that the item had been asked in the GSS in at least three different years with a time span of at least 8 years from the first to the last year. This resulted in a selection of 98 items from the GSS, listed in Supplementary Table 1.

#### Sample and Procedure

Participants in the United States were recruited through Mturk. Prescreening was used to invite equal numbers of self-identified conservatives and liberals and equal numbers of persons who scored higher (eight or higher) and lower (seven or lower) on the Wordsum test of verbal ability (the threshold is based on median in the prescreening sample). The final sample consisted of 568 participants with a mean age of 39.2 years (SD = 12.0) and a fairly balanced composition with respect to gender (59% women, 41% men), political identity (53% liberals, 47% conservatives), verbal ability (48% higher, 52% lower), and education (53% higher, 47% lower). Participants were presented with a series of moral opinions drawn in random order from the 98 GSS items. The participant could stop at any time; the average participant responded to 19 items. Every item was thereby judged by 110 participants on average (Note that we do not need every participant to respond to every item because our research question is posed at the population level: Will a given argument be judged as applicable to a given moral opinion equally often in the male and female populations, equally often among liberals as among conservatives, etc.?).

The procedure for each item was as follows. An item was presented (e.g., “Do you favor the death penalty for persons convicted of murder?”). The participant gave their answer using a dichotomous response scale (yes/no) and was then given the following instructions: “Now consider why you chose that answer. Which of the following arguments apply? Please tick all that apply.” For each item the presented list of arguments consisted of a random draw of one argument of each kind from the list in Table 1, plus “some other reason.” Participants could choose any number of arguments from this list. Arguments were worded to match whether the participant's answer had been yes or no (e.g., “Yes, because otherwise someone is denied his or her rights” or “No, because then someone is denied his or her rights”). Finally, the participant was asked for the arguments they expected to be chosen by someone who had given the *opposite* answer to the item. The same selection of arguments, but reworded to match the opposite answer, was presented for the participant to choose from. Thus, every participant chose arguments for both sides on the issue.

#### Analysis

In a given population, define the *argument applicability score A*_*mc,isspos*_ as the proportion of individuals who think that arguments based on a given moral concern *mc* apply to a given issue position *isspos*. In practice, we estimate this proportion by the corresponding proportion in the sample of the population who responded to the corresponding item. For a fixed moral concern we expect argument applicability scores to strongly depend on the issue position. Similarly, for a fixed issue position we expect argument applicability scores to strongly depend on the moral concern.

After measuring the applicability of the selected set of moral concerns on the selected set of moral issues in samples of different populations, we need to quantify the extent to which two populations agree with each other. Let $A_{\text{mf},\text{isspos}}^{\left(1\right)}$ and $A_{\text{mf},\text{isspos}}^{\left(2\right)}$ denote the estimated argument applicability scores in populations 1 and 2, respectively. If we plot these scores against each other, perfect agreement would yield a perfect line with a 45 degree slope through the origin (y = x). To measure deviations from linearity one can use the Pearson correlation, but it will not detect deviation from perfect agreement in terms of slope or intercept. Instead we will use the concordance correlation coefficient, CCC for short (Lin, 1989). The CCC is calculated as the covariance of the two groups' scores divided by the sum of each group's variance and the square of the difference between their mean scores. The CCC takes values between −1 and 1, and the absolute value of the CCC is always less than or equal to the absolute value of the Pearson correlation. The CCC is very similar to population intraclass correlation coefficients (Nickerson, 1997), but perhaps more intuitive.

Our null hypothesis is that there is no difference between populations in argument applicability. In other words, if we had access to argument applicability scores for the entire populations the null hypothesis is that they would be in perfect agreement (CCC = 1). The observed agreement between samples would still be less than perfect, however, due to sampling error. Under the null hypothesis, the two samples can be regarded as random draws from the same population. To test this we pool the samples from the two populations. We repeatedly (1,000 times) simulate a random split of the pooled sample into two subsamples (of the same size as the original samples) and observe the agreement (CCC) between the argument applicability scores obtained in the two subsamples. We report the *expected observed agreement under perfect true agreement* as the mean observed CCC across 1,000 simulated random splits. This number, rather than 1, is the proper reference value representing perfect agreement. A *p*-value for the null hypothesis that the populations are in perfect agreement is obtained by calculation of the proportion of simulations that have a lower CCC value than the one observed for the actual samples.

### Results and Discussion

**Figure 1:**
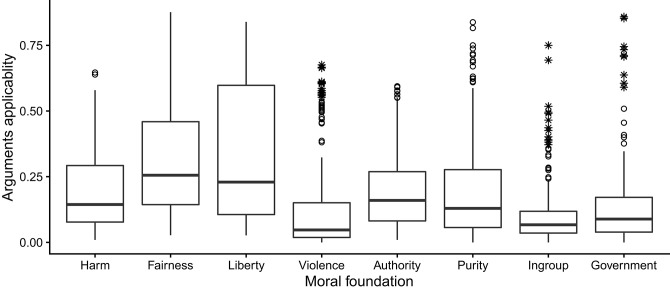
Boxplots showing how applicability, estimated in the entire sample of Study 1, of eight different kinds of moral arguments varied across 196 moral opinions. The box represents the interquartile (IQ) range with the dark line indicating the median. The whiskers reach the min and max values in case these are at most 1.5 times the box height outside the IQ range. Circles and stars signify outliers (values between 1.5 and three times the IQ range) and extreme outliers (more than three times the IQ range), respectively.

Based on the entire sample of participants, the boxplots in Figure 1 show how the applicability scores of different kinds of moral arguments varied across 196 moral opinions (two positions on each of 98 moral questions). Note that every kind of argument had at least some applicability scores well above 0.50. In other words, for every kind of argument there were some opinions that a majority of participants agreed the argument applies to. Thus, the arguments we study are indeed in use. Although not a focus of the present study, it is noteworthy that some arguments seem to be used more often than others. The median applicability (the dark line in each box) was highest for fairness and liberty arguments. Thus, fairness and liberty arguments may be more generally applicable than other kinds of moral arguments, at least for the moral issues included in the GSS.

**Figure 2:**
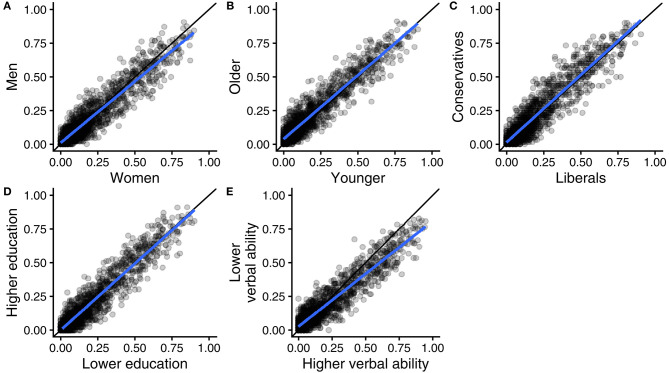
Scatter plots of 1,568 argument applicability scores (eight types of arguments by 196 moral opinions), measured in different groups in the United States: women vs. men **(A)**, younger vs. older **(B)**, liberals vs. conservatives **(C)**, higher vs. lower education **(D)**, and higher vs. lower verbal ability **(E)**. Regression lines in blue and reference lines for perfect agreement in black.

We are interested in the extent to which different groups agree on how the applicability of arguments varies across opinions. Figure 2 uses scatter plots to show how different groups rated each opinion on the applicability of different arguments, illustrating the agreement between samples of women and men (panel A), between samples of younger and older people (panel B), between samples of liberals and conservatives (panel C), between samples of people with higher vs. lower education (panel D), and between samples of people with higher vs. lower verbal ability (panel E). Every dot in a plot refers to the applicability of a specific kind of argument to a specific opinion (e.g., the applicability of fairness arguments to justify favoring the death penalty for murderers), measured in two different subsamples. As the study comprises eight different kinds of arguments and 196 different opinions, there are 8 × 196 = 1,568 dots in each scatter plot. The x-axis and y-axis refer to applicability scores obtained in two different subsamples (e.g., women vs. men).

Perfect agreement between the subsamples would be represented by all dots laying on the 45 degree line through the origin (drawn in black). However, because our subsamples are of limited size, there will inevitably be some sampling error. With perfect agreement at population level between the sampled groups, the expected observed agreement under perfect true agreement is 0.94. As reported in Table 2, the CCC values we observed were extremely close to 0.94. In two cases (sex and education) the null hypothesis of perfect agreement between populations could not be rejected, while for the other cases we must conclude that agreement is not perfect but very nearly perfect.

**Table 2:** CCC values with 95% confidence interval.

**Compared subsamples**	**CCC with 95% CI**	**Expected CCC based on random split**	***p***
Women vs. men	0.94 [0.93, 0.94]	0.940	0.385
Younger vs. older	0.94 [0.93, 0.94]	0.942	0.009
Liberals vs. conservatives	0.93 [0.92, 0.93]	0.941	0.000
Higher vs. lower education	0.95 [0.94, 0.95]	0.940	0.995
Higher vs. lower verbal ability	0.92 [0.91, 0.92]	0.942	0.000

*A p-value of 0.000 means that none of the 1,000 simulated random splits yielded a CCC value as low as the observed one*.

In addition to the 45 degree line through the origin, each scatter plot in Figure 2 includes the regression line that best fits the data. The two lines are typically very close to each other, meaning that there was no systematic disagreement between the two groups that were compared. Instead the observed disagreement was chiefly non-systematic, in the form of “noise” around the regression line that inevitably comes with sampling error. Similar results were obtained with individual-level logistic regressions, performed separately for each combination of a specific moral opinion and a specific moral concern. Supplementary Figures 1, 2 show that strong effects of individual characteristics and their interactions are rare and mostly within what would be expected by chance.

Note, however, that the slope of the regression line in Panel E is noticeably <45 degrees. This indicates that ratings of the applicability of arguments for different moral opinions were less distinct in the lower verbal ability sample (on the y-axis) than in the higher verbal ability sample (on the x-axis). We therefore examined the variance in ratings of different moral opinions and found that it was consistently smaller in the lower ability sample than in the higher ability sample; the variance ratio between the two subsamples was well below 1 for all types of arguments, ranging from 0.52 (ingroup) to 0.73 (government). We return to this observation in the general discussion.

In conclusion, Study 1 demonstrated two important things about the applicability of moral arguments. First, each kind of moral argument applied strongly to certain moral opinions, while applying weakly to certain other moral opinions, and not at all to some opinions. Second, to measure this variation in argument applicability across opinions, it did not matter who we asked; different populations were in near-perfect agreement.

## Study 2

### Method

#### Selection of Moral Questions in the United Kingdom

Similar to the GSS in the United States, the United Kingdom has an annual survey to representative samples of the British population called the British Social Attitudes survey, abbreviated BSA (NatCen Social Research, 2019). Application of the same inclusion criteria and procedure as used for the GSS resulted in a selection of 108^1^ items from the BSA, listed in Supplementary Table 2.

#### Sample and Procedure

Through Prolific we recruited 903 participants of UK nationality, with a mean age of 38.3 years (SD = 12.8). Unlike Study 1, we did not do our own prescreening of participants (hence, no measure of verbal ability was collected). Instead we relied on Prolific's demographical data to obtain a sample covering the entire political spectrum from left to right. On an 11 point scale from political left to right, 25% of participants identified as left-wing (0–3), 41% as moderate (4–6), and 34% as right-wing (7–10). The sample also had a fairly balanced composition with respect to gender (58% women, 42% men) and education (52% higher, 48% lower). Participants were rewarded £0.10 per BSA item they judged. Every item was judged by 103 participants on average. The procedure was the same in Study 1.

### Results and Discussion

**Figure 3:**
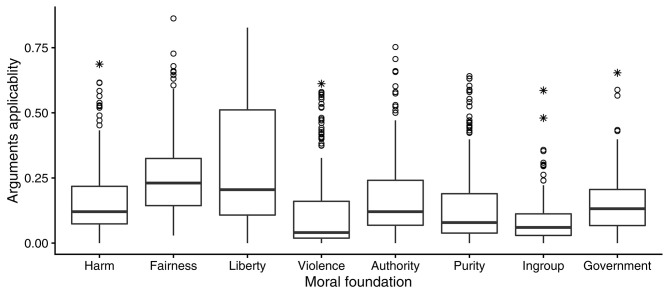
Boxplots (defined in Figure 2) showing how applicability, estimated in the entire sample of Study 2, of eight different kinds of moral arguments varied across 216 moral opinions.

Results in the United Kingdom closely replicated the results in the United States in Study 1. To begin with, every argument was strongly applicable to at least some opinions (see Figure 3). Also like in Study 1, fairness and liberty were the most generally applicable kinds of arguments.

**Figure 4:**
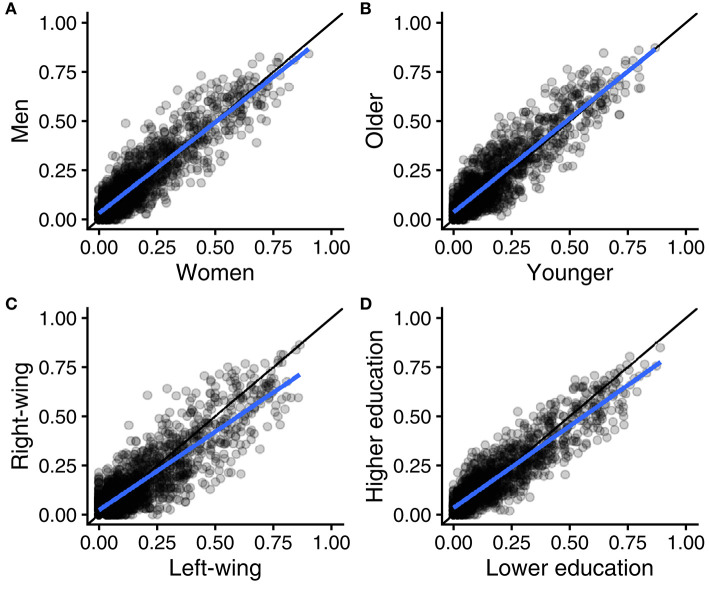
Scatter plots of 1,728 argument applicability scores (eight types of arguments by 216 moral opinions), measured in different groups in the United Kingdom: women vs. men **(A)**, younger vs. older **(B)**, right-wing vs. left-wing **(C)**, and higher vs. lower education **(D)**. Regression lines in blue and reference lines for perfect agreement in black.

Study 2 also replicated the finding of near-perfect agreement between women and men, between younger and older people, between left-wing and right-wing, and between people with higher vs. lower education (see Figure 4, Supplementary Figures 3, 4). As reported in Table 3, CCC values ranged from 0.86 to 0.91, with 0.91 equalling the expected value under perfect agreement at population level.

**Table 3:** The CCC value with 95% confidence interval.

**Compared subsamples**	**CCC with 95% CI**	**Expected CCC based on random split**	***p***
Women vs. men	0.90 [0.89, 0.91]	0.917	0.000
Younger vs. older	0.89 [0.88, 0.90]	0.919	0.000
Left-wing vs. right-wing	0.86 [0.85, 0.87]	0.874	0.011
Higher vs. lower education	0.91 [0.90, 0.92]	0.918	0.001

*A p-value of 0.000 means that none of the 1,000 simulated random splits yielded a CCC value as low as the observed one*.

## Study 3

The aim of the third study is to examine the extent of cross-cultural agreement on moral argument applicability. In addition to the United States and the United Kingdom, we include two more culturally distant societies: Israel and Brazil.

### Method

#### Selection of Moral Questions in Israel and Brazil

The selections of moral questions in the US and UK had an overlap of 27 questions, listed in Supplementary Table 3. For the study in Israel and Brazil we used these 27 questions in the US version, replacing “America” by “Israel” or “Brazil” where applicable.

#### Sample and Procedure

Through Prolific we recruited 223 participants of Israeli nationality who are also residents in Israel, with a mean age of 28.8 years (SD = 9.3). On an 11 point scale from political left to right, 24% identified as left-wing (0–3), 45% as moderate (4–6), and 31% as right-wing (7–10). Women constituted 52% of the sample. Participants were rewarded £0.15 per item they judged. Every item was judged by 101 participants on average. The procedure was the same in Study 1, yielding argument applicability ratings of 54 moral opinions in Israel.

Regarding the Brazilian sample, through Facebook's “Boost post” tool we recruited 294 individuals of Brazilian nationality who reside in Brazil, with a mean age of 44.2 years (SD = 13.9). On an 11 point scale from political left to right, 48% identified as left-wing (0–3), 28% as moderate (4–6), and 24% as right-wing (7–10). Women constituted 44% of the sample. In contrast to Israeli participants, Brazilian ones were not paid to judge items. Instead, we added feedback at the end of the survey as motivation for them to answer it–through such feedback they could assess how many participants have the same moral opinions as them, and how many have different ones. Each participant judged nine randomly selected items. Every item was judged by 98 participants on average. The rest of the procedure was the same in Study 1, yielding argument applicability ratings of 54 moral opinions in Brazil.

### Results

**Figure 5:**
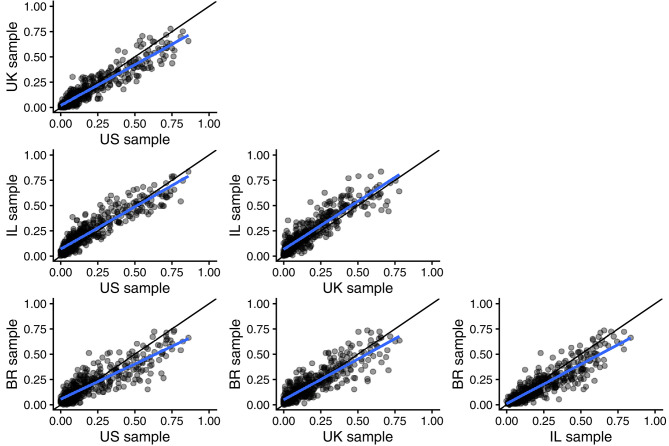
Scatter plots of 432 argument applicability scores for (eight types of arguments by 54 moral opinions). Each row compares data from two of our four countries US, UK, Israel (IL), and Brazil (BR). Regression lines in blue and reference lines for perfect agreement in black.

Figure 5 presents scatter plots of argument applicability scores for the 54 moral opinions in the United States (data from Study 1) and in the United Kingdom (data from Study 2), as well as in Israel and Brazil (data from Study 3). The agreement between the four different countries was overall very high. The CCC values were just slightly lower than the expected CCC under perfect agreement on the population level, see Table 4.

**Table 4:** The CCC value with 95% confidence interval.

**Compared subsamples**	**CCC with 95% CI**	**Expected CCC based on random split**	***p***
US UK	0.92 [0.90, 0.93]	0.965	0.000
US IL	0.90 [0.88, 0.92]	0.962	0.000
US BR	0.85 [0.82, 0.87]	0.958	0.000
UK IL	0.86 [0.83, 0.88]	0.955	0.000
UK BR	0.87 [0.84, 0.89]	0.951	0.000
IL BR	0.85 [0.82, 0.87]	0.948	0.000

### Agreement on Argument Applicability Between People Who Agree vs. Disagree With the Opinion

So far we have examined whether different demographic groups agree on how the applicability of moral arguments varies across different moral opinions. However, our data also include participants' own opinion on each issue. Within every demographic group there will be some diversity in opinion on any issue, and this diversity will not be aligned across different issues (i.e., two individuals with agreeing opinions on one issue may have disagreeing opinions on another issue). To split the sample based on their opinions, we therefore need to make a separate split for each moral issue. Because some opinions are unusual, some subsamples will then be small, thereby increasing sampling error and hence decreasing the expected CCC. The results of this analysis is presented in Table 5, showing that people with opposing opinions still showed nearly perfect agreement on applicable arguments in the United States and the United Kingdom, and only slightly less agreement in Israel and Brazil.

**Table 5:** CCC values, with 95% confidence intervals, measuring in four countries the extent to which people with different opinions on the underlying moral issues agreed on argument applicability.

**Country**	**Number of opinions**	**CCC with 95% CI**	**Expected CCC under perfect agreement**	***p***
United States	194	0.82 [0.80, 0.83]	0.853	0.021
United Kingdom	216	0.73 [0.71, 0.75]	0.782	0.034
Israel	54	0.57 [0.50, 0.63]	0.692	0.017
Brazil	52	0.53 [0.46, 0.60]	0.723	0.014

*In Brazil the number of opinions is 52 instead of 54 because we had to exclude one item for which all participants had the same opinion*.

## General Discussion

In this paper we have examined whether there are “objective” measures of how specific moral concerns apply to specific moral opinions. In support of this notion, we found generally very high levels of agreement between applicability scores obtained from different subsamples. It is perhaps unremarkable that there was agreement between men and women or between younger and older people, but we also compared groups with different ideologies: liberals and conservatives. Moral foundations research has found that liberals and conservatives recognize different kinds of arguments as relevant to their own moral judgments (Graham et al., 2011). It has further been argued that this difference accounts for ideological differences in opinions on moral issues (Koleva et al., 2012). The validity of the latter theory rests on liberals and conservatives agreeing on which arguments apply to each side of an issue, so that they can choose their opinion based on which of these arguments are most relevant to them personally. In support of this theory, we found that liberals and conservatives generally agreed on the applicability of arguments.

We further compared subsamples based on verbal ability, which is known to be an important predictor of political attitudes (Ludeke et al., 2017). We found that respondents with high vs. low verbal ability generally agreed on the applicability of moral arguments. However, there was still a notable difference between the groups with respect to how much they distinguished between different moral opinions when rating the applicability of arguments. Specifically, it was the low ability group that made less distinctions. Given that our overall findings indicate an objective connection between specific opinions and specific arguments, the finding on verbal ability suggests that people with lower verbal ability tend to have a somewhat less clear understanding of this connection.

So far, we have discussed fixed groupings. In an additional analysis we also examined groupings based on opinions. Such groupings vary across different moral issues because any given individual is sometimes for and sometimes against, depending on the issue. However, even in this analysis we found relatively high agreement. In other words, the arguments used to justify a given opinion tend to be recognized even by those who hold the opposite opinion themselves.

Finally, we observed general agreement across samples from the United States, the United Kingdom, Israel, and Brazil—countries located in four different continents. Although it is possible that there are yet other cultures that do not agree, the cross-cultural invariance observed in this study at least suggests that the applicability of moral arguments to moral opinions has a partly objective component and is not simply a cultural convention.

### Implications

According to the moral argument theory of opinion dynamics (Eriksson and Strimling, 2015), exchange of arguments may be an important mechanism for population-level opinion change. Even though listening to another's argument will most often not change the listener's opinion, it is enough that individual change happens sometimes, and more often in one direction than the other, for a noticeable trend to emerge at the population level. Prior research indicates that measures of argument applicability can then be used to predict which position on a given issue is associated with liberals and in which direction public opinion is trending (Strimling et al., 2019). Our findings indicate that such measures can be trusted even if they are based on convenience samples.

### Limitations

Our study is based on samples of demographic groups, samples of moral arguments, and samples of moral issues. It is prudent to consider whether our conclusions are likely to generalize beyond these samples. A potential source of bias is that our sample of participants were recruited among users of Mturk or Prolific. However, it is unlikely that this factor would matter for their ratings of moral arguments when more fundamental individual properties such as opinions, ideology, cognitive ability, and nationality mattered so little. With regards to the sample of moral arguments, we expanded it to 24 arguments from the 15 used in a previous study. As we find that all 24 arguments were applied similarly across groups this is likely to generalize to other arguments not covered so far. Finally, our sample of issues included all moral issues covered by two large surveys in the United States and the United Kingdom. This selection is probably biased toward controversial issues, but it seems unlikely that issues on which there is consensus in the population would yield less agreement on argument applicability.

Our observation of cross-cultural agreement on argument applicability was based on only four countries. While suggestive of a broader pattern of cross-cultural agreement, its scope will not be known until data are available from a greater range of different cultures.

A potential problem with our study design is that we provide participants with arguments to choose from, thereby possibly initiating a reflection on arguments that they would otherwise not have thought of. An alternative method would be to use free-text questions and have responses coded for the different moral concerns. It would be interesting to see whether this would lead to any qualitative change in argument applicability scores.

### Conclusion

This study sheds light on the level of subjectivity of the moral mind. People with different moral opinions do not seem to live in different moral universes. We found that the justifications people give for their opinions are specific to each moral issue and that others who do not share their opinion recognize these specific justifications. Thus, it seems like the disagreement on moral issues is not based on how various moral opinions may be justified but rather which of the justifications are most important.

## Data Availability Statement

The data and R code to reproduce the results of the paper are available at https://github.com/irinavrt/moral-args-appli.

## Ethics Statement

Ethical review and approval was not required for the study on human participants in accordance with the local legislation and institutional requirements. The patients/participants provided their written informed consent to participate in this study.

## Author Contributions

PS, KE, and IV conceived and designed the study. IV and IH collected the data. IV performed all analyses with input from KE. KE drafted the manuscript. All authors gave critical input on and approved the manuscript.

## Conflict of Interest

The authors declare that the research was conducted in the absence of any commercial or financial relationships that could be construed as a potential conflict of interest.

## Footnotes

^1^An item about availability of pornographic material had response options ranging from “should be banned altogether” to “should be available in any shop for sale to anyone” with intermediate options describing various restrictions on which shops and to whom. For argument collection and analysis we split this item into two: whether or not the sale of pornography should be banned altogether, and whether or not the sale of pornography to adults should be allowed in any shop.
