# Supplementary material for: Different Populations Agree on Which Moral Arguments Underlie Which Opinions
Source: Front Psychol. 2021 Mar 15;12:648405. doi: 10.3389/fpsyg.2021.648405 (PMC8005634; doi:10.3389/fpsyg.2021.648405)
Supplement: Supplementary file 1 [file Image_1.PDF]

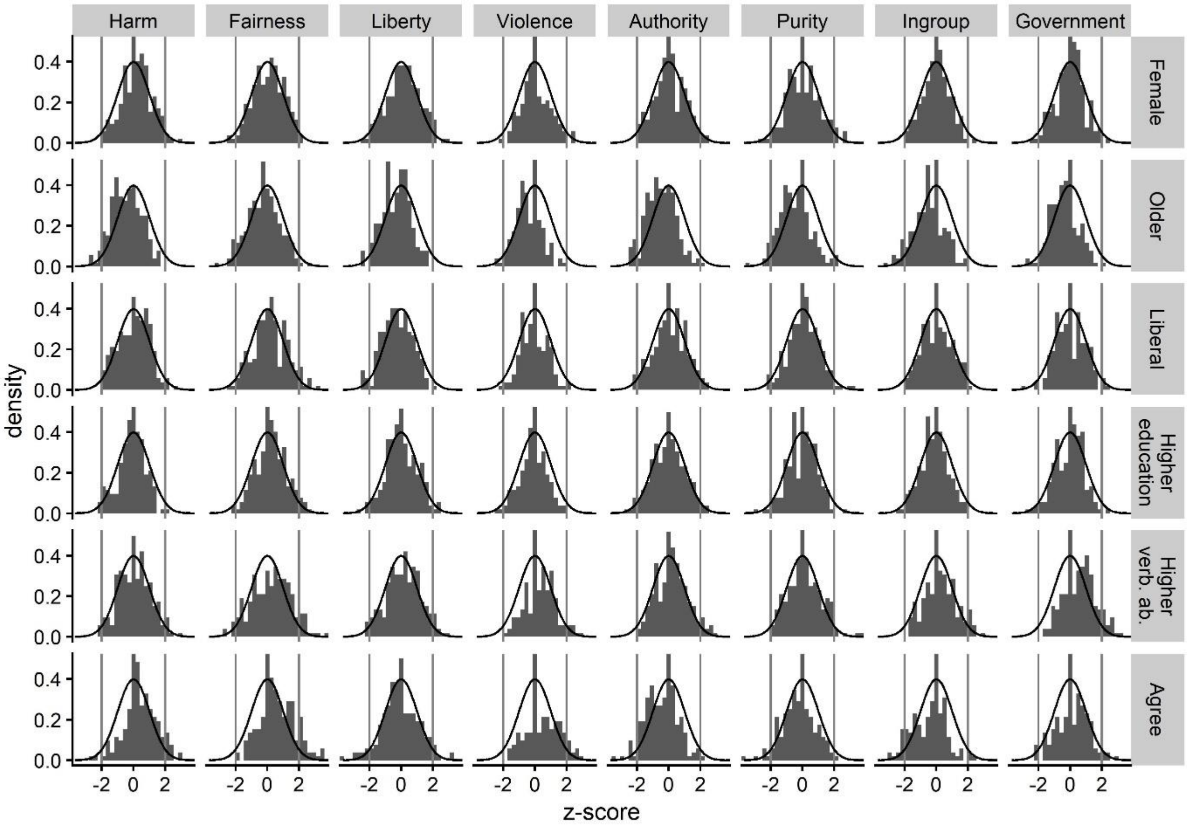

Supplementary Figure 1. We use logistic regression to explore the marginal effects of individual characteristics controlling for rater's own opinion as well their interactions. The histograms show distributions of Z-scores for the effect of sex, age, ideology, education, verbal ability, and agreement with the opinion on the probability to find each of the eight kinds of argument applicable to each of the 196 GSS opinions in the US sample. For comparison, the black line shows the standard normal distribution. The vertical grey lines separate coefficients that are above or below zero by at least two standard errors. The y-axis scale is cut at 0.5 for better visibility, otherwise the bar representing the zero effect sometimes reach as high as 2.
