# Supplementary material for: Different Populations Agree on Which Moral Arguments Underlie Which Opinions
Source: Front Psychol. 2021 Mar 15;12:648405. doi: 10.3389/fpsyg.2021.648405 (PMC8005634; doi:10.3389/fpsyg.2021.648405)
Supplement: Supplementary file 2 [file Image_2.PDF]

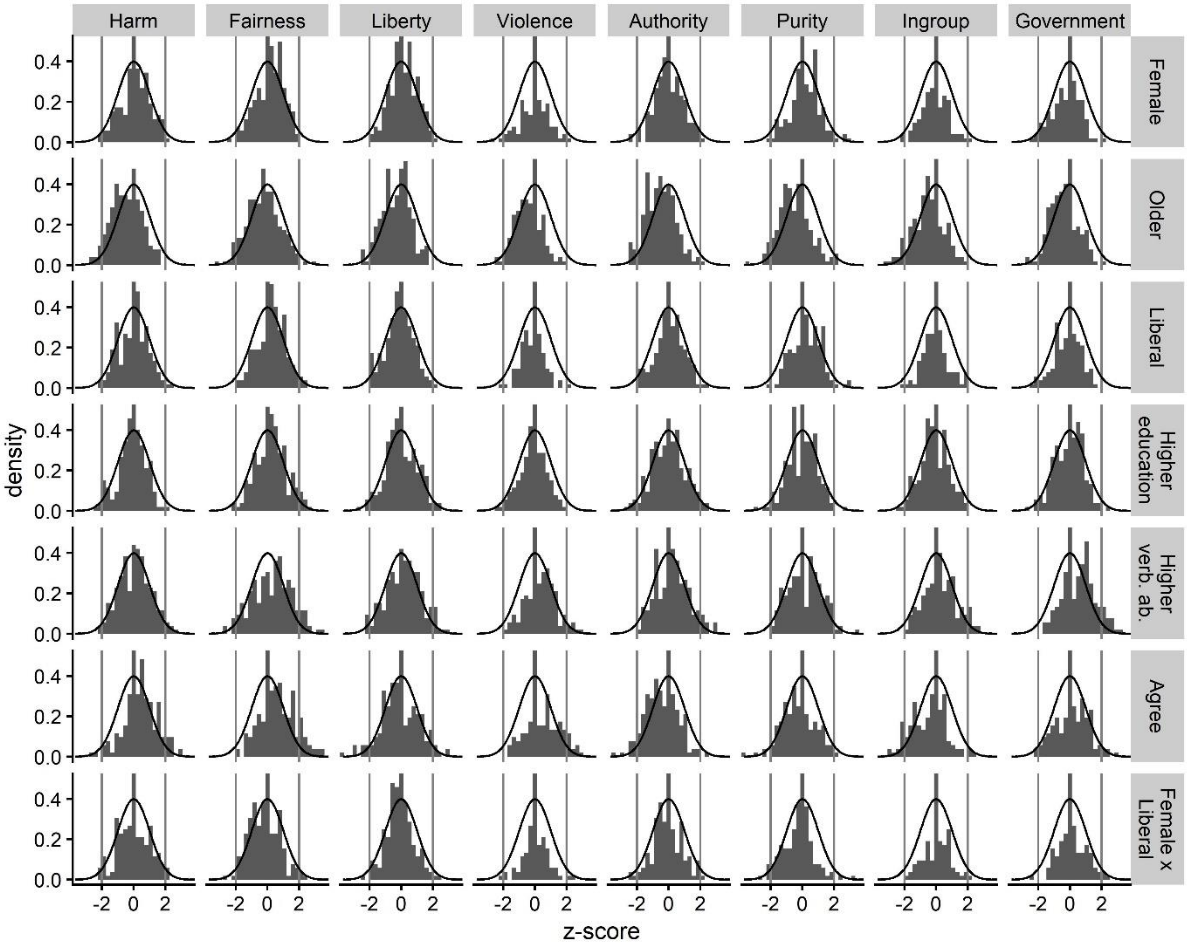

Supplementary Figure 2. The same as Supplementary Figure 1 except it also includes estimates of the interaction between gender and ideology. Although not presented here, similar results were obtained for the interaction between ideology and other individual characteristics.
