# Supplementary material for: Different Populations Agree on Which Moral Arguments Underlie Which Opinions
Source: Front Psychol. 2021 Mar 15;12:648405. doi: 10.3389/fpsyg.2021.648405 (PMC8005634; doi:10.3389/fpsyg.2021.648405)
Supplement: Supplementary file 4 [file Image_4.PDF]

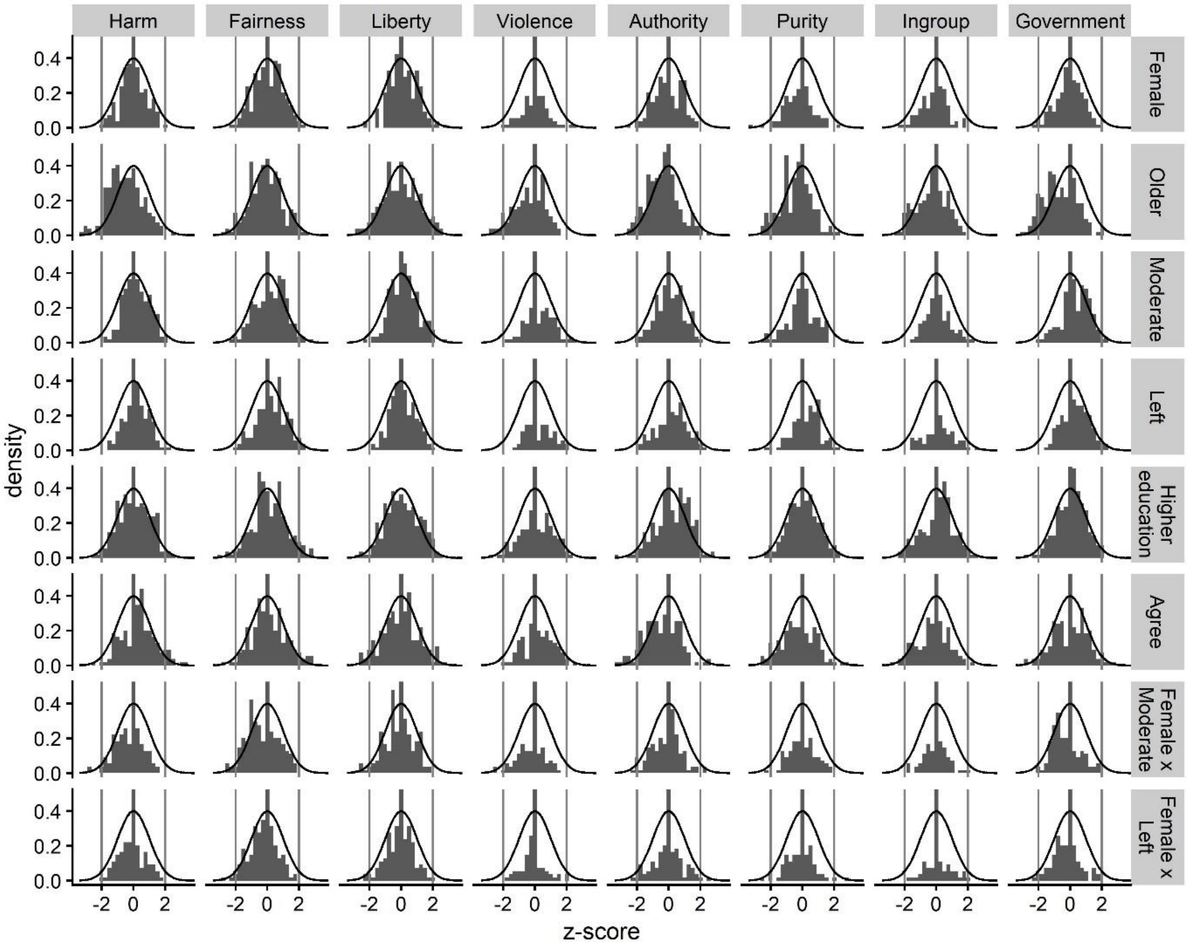

Supplementary Figure 4. The same as Supplementary Figure 3, except it also includes estimates of the interaction between gender and ideology. Although not presented here, similar results were obtained for the interaction between ideology and other individual characteristics.
