# Supplementary material for: Different Populations Agree on Which Moral Arguments Underlie Which Opinions
Source: Front Psychol. 2021 Mar 15;12:648405. doi: 10.3389/fpsyg.2021.648405 (PMC8005634; doi:10.3389/fpsyg.2021.648405)
Supplement: Supplementary file 5 [file Table_1.DOCX]

**Supplementary Table 1.** The list of 98 GSS moral items.

| **Question** | **GSS variable name** |
| --- | --- |
| Do you think it should be possible for a pregnant woman to obtain a legal abortion if the woman wants it for any reason? | abany |
| Do you personally think it is wrongfor a woman to have an abortion if there is a strong chance of serious defect in the baby. | abdefctw |
| Do you think it should be possible for a pregnant woman to obtain a legal abortion if there is a strong chance of serious defect in the baby? | abdefect |
| Do you think it should be possible for a pregnant woman to obtain a legal abortion if the woman’s own health is seriously endangered by the pregnancy? | abhlth |
| Do you think it should be possible for a pregnant woman to obtain a legal abortion if she is married and does not want any more children? | abnomore |
| Do you think it should be possible for a pregnant woman to obtain a legal abortion if the family has a very low income and cannot afford any more children? | abpoor |
| Do you personally think it is wrong for a woman to have an abortion if the family has a very low income and cannot afford any more children. | abpoorw |
| Do you think it should be possible for a pregnant woman to obtain a legal abortion if she became pregnant as a result of rape? | abrape |
| Do you think it should be possible for a pregnant woman to obtain a legal abortion if she is not married and does not want to marry the man? | absingle |
| Even if it brings no immediate benefits - scientific research that advances the frontiers of knowledge is necessary and should be supported by the federal government. | advfront |
| Some people say that because of past discrimination - blacks should be given preference in hiring and promotion. Others say that such preference in hiring and promotion of blacks is wrong because it discriminates against whites. What about your opinion - are you for preferential hiring and promotion of blacks? | affrmact |
| America should follow its own interests - even if this leads to conflicts with other nations. | amownway |
| American television should give preference to American films and programs. | amtv |
| Government should let businesses decide for themselves how to protect the environment - even if it means they don’t always do the right thing - rather than passing laws to make businesses protect the environment as it interferes with business’ right to make their own decisions. | busdecid |
| In general: do you favor the busing of African-American and white school children from one school district to another? | busing |
| Do you favor the death penalty for persons convicted of murder? | cappun |
| Those in need have to learn to take care of themselves and not depend on others. | careself |
| Religious leaders should not try to influence government decisions. | clerggov |
| Religious leaders should not try to influence how people vote in elections. | clergvte |
| There are always some people whose ideas are considered bad or dangerous by other people. For instance: somebody who is against all churches and religion – should such a person be allowed to teach in a college or university? | colath |
| Consider a man who admits he is a Communist. Suppose he is teaching in a college. Should he be fired? | colcom |
| Consider a man who admits that he is a homosexual. Should such a person be allowed to teach in a college or university? | colhomo |
| Consider a person who advocates doing away with elections and letting the military run the country. Should such a person be allowed to teach in a college or university? | colmil |
| Now consider a Muslim clergyman who preaches hatred of the United States. Should such a person be allowed to teach in a college or university? | colmslm |
| Consider a person who believes that Blacks are genetically inferior. Should such a person be allowed to teach in a college or university? | colrac |
| It is much better for everyone involved if the man is the achiever outside the home and the woman takes care of the home and family. | fefam |
| It is more important for a wife to help her husband’s career than to have one herself. | fehelp |
| Because of past discrimination employers should make special efforts to hire and promote qualified women. | fehire |
| Women should take care of running their homes and leave running the country up to men. | fehome |
| Some people say that because of past discrimination women should be given preference in hiring and promotion. Others say that such preference in hiring and promotion of women is wrong because it discriminates against men. What about your opinion - are you for preferential hiring and promotion of women? | fejobaff |
| Do you approve of a married woman earning money in business or industry if she has a husband capable of supporting her? | fework |
| Foreigners should not be allowed to buy land in America. | forland |
| Do you think the use of marijuana should be made legal? | grass |
| On the whole - do you think it should be the government’s responsibility to impose strict laws to make industry do less damage to the environment? | grnlaws |
| Would you favor a law which would require a person to obtain a police permit before he or she could buy a gun? | gunlaw |
| Some people think that African-Americans have been discriminated against for so long that the government has a special obligation to help improve their living standards. Others believe that the government should not be giving special treatment to African-Americans. Do you think that the government should help improve the living standards of African-Americans? | helpblk |
| Some people think that the government in Washington should do everything possible to improve the standard of living of all poor Americans. Other people think it is not the government’s responsibility and that each person should take care of himself. Do you think that the government should do everything possible to improve the standard of living of all poor Americans? | helppoor |
| In general some people think that it is the responsibility of the government in Washington to see to it that people have help in paying for doctors and hospital bills. Others think that these matters are not the responsibility of the federal government and that people should take care of these things themselves. Do you think that it is the responsibility of the government? | helpsick |
| Would you approve of a man punching a stranger who was beating up a woman and the man saw it? | hitbeatr |
| Would you approve of a man punching a stranger who had hit the man’s child after the child accidentally damaged the stranger’s car? | hitchild |
| Would you approve of a man punching a stranger who was drunk and bumped into the man and his wife on the street? | hitdrunk |
| Would you approve of a man punching a stranger who was in a protest march showing opposition to the other man’s views? | hitmarch |
| Are there any situations that you can imagine in which you would approve of a man punching an adult male stranger? | hitok |
| Would you approve of a man punching a stranger who had broken into the man’s house? | hitrobbr |
| What about sexual relations between two adults of the same sex–do you think it is always wrong? | homosex |
| A man’s job is to earn money - a woman’s job is to look after the home and family. | hubbywk1 |
| People should support their country even if the country is in the wrong. | ifwrong |
| When a person has a disease that cannot be cured – do you think doctors should be allowed by law to end the patient’s life by some painless means if the patient and his family request it? | letdie1 |
| There are always some people whose ideas are considered bad or dangerous by other people. For instance somebody who is against all churches and religion. If some people in your community suggested that a book he wrote against churches and religion should be taken out of your public library – would you favor removing this book? | libath |
| Consider a man who admits he is a Communist. Suppose he wrote a book which is in your public library. Somebody in your community suggests that the book should be removed from the library. Would you favor removing it? | libcom |
| Consider a man who admits that he is a homosexual. If some people in your community suggested that a book he wrote in favor of homosexuality should be taken out of your public library – would you favor removing this book? | libhomo |
| Consider a person who advocates doing away with elections and letting the military run the country. Suppose he wrote a book advocating doing away with elections and letting the military run the country. Somebody in your community suggests that the book be removed from the public library. Would you favor removing it? | libmil |
| Consider a person who believes that Blacks are genetically inferior. If some people in your community suggested that a book he wrote which said Blacks are inferior should be taken out of your public library – would you favor removing this book? | librac |
| Would you be in favor of having a close relative or family member marry an Asian American person? | marasian |
| Would you be in favor of having a close relative or family member marry a black person? | marblk |
| Would you be in favor of having a close relative or family member marry a Hispanic American person? | marhisp |
| Homosexual couples should have the right to marry one another. | marhomo |
| People who want children ought to get married. | marlegit |
| Would you be in favor of having a close relative or family member marry a white person? | marwht |
| Ethnic minorities should be given government assistance to preserve their customs and traditions. | mincult |
| In general - would you say that people should obey the law without exception rather than there are exceptional occasions on which people should follow their consciences even if it means breaking the law? | obeylaw |
| People should be willing to help others who are less fortunate. | othshelp |
| Do you agree that methods of birth control should be available to teenagers between the ages of 14 and 16 if their parents do not approve? | pillok |
| Would you approve of a policeman striking a citizen who had said vulgar and obscene things to the policeman? | polabuse |
| Would you approve of a policeman striking a citizen who was attacking the policeman with his fists? | polattak |
| Would you approve of a policeman striking a citizen who was attempting to escape from custody? | polescap |
| Are there any situations you can imagine in which you would approve of a policeman striking an adult male citizen? | polhitok |
| Would you approve of a policeman striking a citizen who was being questioned as a suspect in a murder case? | polmurdr |
| Consider your feelings about pornography laws. Do you think it should be illegal? | pornlaw |
| There’s been a lot of discussion about the way morals and attitudes about sex are changing in this country. If a man and woman have sex relations before marriage – do you think it is wrong? | premarsx |
| Government should let ordinary people decide for themselves how to protect the environment - even if it means they don’t always dothe right thing - rather than passing laws to make ordinary people protect the environment as it interferes with people’s right to make their own decisions. | pubdecid |
| Those who violate God’s rules must be punished. | punsin |
| If you and your friends belonged to a social club that would not let African-Americans join – would you try to change the rules so that African-Americans could join? | racchng |
| Would you yourself have any objection to sending your children to a school where a few of the children are African-Americans? | racfew |
| Would you yourself have any objection to sending your children to a school where half of the children are African-Americans? | rachaf |
| Do you think there should be laws against marriages between African-Americans and whites? | racmar |
| Would you yourself have any objection to sending your children to a school where more than half of the children are African-Americans? | racmost |
| Suppose there is a community-wide vote on the general housing issue. There are two possible laws to vote on (OWNER DECIDES and CAN’T DISCRIMINATE). Would you vote for owner decides? | racopen |
| African-Americans shouldn’t push themselves where they’re not wanted. | racpush |
| White people have a right to keep African-Americans out of their neighborhoods if they want to and African-Americans should respect that right. | racseg |
| Would you be for sex education in the public schools? | sexeduc |
| Do you agree that it is sometimes necessary to discipline a child with a good hard spanking? | spanking |
| Consider somebody who is against all churches and religion. If such a person wanted to make a speech in your (city/town/community) against churches and religion – should he be allowed to speak? | spkath |
| Consider a man who admits he is a Communist. Suppose this admitted Communist wanted to make a speech in your community. Should he be allowed to speak? | spkcom |
| Consider a man who admits that he is a homosexual? Suppose this admitted homosexual wanted to make a speech in your community. Should he be allowed to speak? | spkhomo |
| Consider a person who advocates doing away with elections and letting the military run the country. If such a person wanted to make a speech in your community – should he be allowed to speak? | spkmil |
| Now consider a Muslim clergyman who preaches hatred of the United States. If such a person wanted to make a speech in your community preaching hatred of the United States - should he be allowed to speak? | spkmslm |
| Consider a person who believes that Blacks are genetically inferior. If such a person wanted to make a speech in your community claiming that Blacks are inferior – should he be allowed to speak? | spkrac |
| Do you think a person has the right to end his or her own life if this person has an incurable disease? | suicide1 |
| Do you think a person has the right to end his or her own life if this person has gone bankrupt? | suicide2 |
| Do you think a person has the right to end his or her own life if this person has dishonored his or her family? | suicide3 |
| Do you think a person has the right to end his or her own life if this person is tired of living and ready to die? | suicide4 |
| Consider people who are in their early teens – say 14 to 16 years old. In that case: do you think sex relations before marriage are wrong? | teensex |
| Both the husband and the wife should contribute to the household income. | twoincs1 |
| Everything considered: would you say that – in general – you approve of wiretapping? | wirtap |
| Irish and Italians and Jewish and many other minorities overcame prejudice and worked their way up. Blacks should do the same without special favors. | wrkwayup |
| For certain problems - like environmental pollution - international bodies should have the right to enforce solutions. | wrldgovt |
| What is your opinion about a married person having sexual relations with someone other than the marriage partner–is it wrong? | xmarsex |
