# Supplementary material for: Different Populations Agree on Which Moral Arguments Underlie Which Opinions
Source: Front Psychol. 2021 Mar 15;12:648405. doi: 10.3389/fpsyg.2021.648405 (PMC8005634; doi:10.3389/fpsyg.2021.648405)
Supplement: Supplementary file 6 [file Table_2.DOCX]

**Supplementary Table 2.** The list of 108 BSA moral items.

| **Question** | **BSA variable name** |
| --- | --- |
| Do you think the law should allow an abortion in case the woman decides on her own she does not wish to have a child. | abort1 |
| Do you think the law should allow an abortion in case the couple agree they do not wish to have the child. | abort2 |
| Do you think the law should allow an abortion in case the woman is not married and does not wish to marry the man. | abort3 |
| Do you think the law should allow an abortion in case the couple cannot afford any more children. | abort4 |
| Do you think the law should allow an abortion in case there is a strong chance of a defect in the baby. | abort5 |
| Do you think the law should allow an abortion in case the woman’s health is seriously endangered by the pregnancy. | abort6 |
| Do you think the law should allow an abortion in case the woman became pregnant as a result of rape. | abort7 |
| Do you personally think it is wrong for a woman to have an abortion if there is a strong chance of serious defect in the baby? | aborwrga |
| Do you personally think it is wrong for a woman to have an abortion if the family has a very low income and cannot afford any more children? | aborwrgb |
| Official warnings about AIDS should say that some sexual practices are morally wrong | aidswrng |
| Do you think schools should or have the legal right to expel children who have AIDS? | aidsxpel |
| An unemployed person on benefit takes a casual job and is paid in cash. He does not report it to the benefit office and is £500 in pocket. Do you feel this is wrong? | ben500 |
| Both the man and woman should contribute to the household income. | bothearn |
| Buses should be given more prionty in towns and cities, even if this makes things more difficult for car dnvers. | busprior |
| People who sell cannabis should always be prosecuted | canselpr |
| People should not be prosecuted for possessing small amounts of cannabis for their own use? | canusepr |
| Are you in favour of the death penalty for murder in the course of a terrorist act? | cappun1 |
| Are you in favour of the death penalty for murder of a police officer? | cappun2 |
| Are you in favour of the death penalty for murder? | cappun3 |
| People should be allowed to use their cars as much as they like, even if it causes damage to the environment. | carallow |
| People who drive cars that are better for the environment should pay less to use the roads than people whose cars are more harmful to the environment. | carenvdc |
| For the sake of the environment everyone should reduce how much they use their cars. | carreduc |
| Censorship of films and magazines is necessary to uphold moral standards. | censor |
| It is acceptible to use animals for testing and improving cosmetics. | cosmtest |
| Suppose the police get an anonymous tip that a man with a long criminal record is planning to break into a warehouse. Do you think the police should be allowed, without a Court Order to keep the man under surveillance? | crimin1 |
| Suppose the police get an anonymous tip that a man with a long criminal record is planning to break into a warehouse. Do you think the police should be allowed, without a Court Order to tap his telephone? | crimin2 |
| Suppose the police get an anonymous tip that a man with a long criminal record is planning to break into a warehouse. Do you think the police should be allowed, without a Court Order to open his mail? | crimin3 |
| Suppose the police get an anonymous tip that a man with a long criminal record is planning to break into a warehouse. Do you think the police should be allowed, without a Court Order to detain the man overnight for questioning? | crimin4 |
| The countryside should be protected from development, even if this sometimes leads to fewer new jobs. | ctryjobs |
| Cyclists and pedestrians should be given more priority in towns and cities even if this makes things more difficult for other road users. | cycpedpr |
| If someone has drunk any alcohol they should not drive. | ddnodrv |
| For some crimes, the death penalty is the most appropriate sentence. | deathapp |
| Older people should be encouraged to retire earlier to reduce unemployment. | earlyret |
| Ethnic minorities should be given government assistance to preserve their customs and traditions. | ethncaid |
| Some people say that it is better for a country if different racial and ethnic groups maintain their distinct customs and traditions. Others say that it is better if these groups adapt and blend into the larger society. Are you in favour of the groups maintaining their traditions? | ethncvw |
| How about a person who is not in much pain nor in danger of death, but becomes permanently and completely dependent on relatives for all their needs - for example someone who cannot feed, wash or go to the toilet by themselves. Do you think that, if they ask for it, a doctor should ever be allowed by law to end their life? | eudepsh |
| A person with an incurable and painful illness, from which they will die - for example someone dying of cancer. Do you think that, if they ask a close relative to end their life, should the law ever allow the close relative to do so? | eurelsh |
| Suppose a person has a painful incurable disease. Do you think that doctors should be allowed by law to end the patient’s life, if the patient requests it? | exit1 |
| What about a married person having sexual relations with someone other than his or her partner, is it always wrong? | exms |
| In times of high unemployment married women should stay at home. | femhome |
| Do you think female homosexual couples - that is lesbians - should be allowed to adopt a baby under the same conditions as other couples? | fgayadpt |
| For certain problems, like environment pollution, international bodies should have the right to enforce solutions. | forgrel2 |
| Britain should follow its own interests, even if this leads to conflicts with other nations. | forgrel4 |
| Foreigners should not be allowed to buy land in Britain. | forgrel5 |
| Britain’s television should give preference to British films and programmes. | forgrel6 |
| Fox hunting should be banned by law. | foxhunt |
| Now I would like you to tell me whether, in your opinion, it is acceptable for a homosexual person to hold a responsible position in public life? | gaypub |
| Now I would like you to tell me whether, in your opinion, it is acceptable for a homosexual person to be a teacher in a college or university? | gayteahe |
| Now I would like you to tell me whether, in your opinion, it is acceptable for a homosexual person to be a teacher in a school? | gayteasc |
| Taking heroin should remain illegal. | herlegal |
| What about sexual relations between two adults of the same sex, is it wrong? | homosex |
| There has been a lot of debate among teachers about how British schools should cater for children whose parents come from other countries and cultures. Do you think in general that schools with many such children should allow those for whom it is important to wear their traditional dress at school? | imchild3 |
| It is more important to keep green-belt areas than to build new homes there. | keepbelt |
| Smoking cannabis (marijuana) should be legalised. | legcan |
| It is all right for a couple to live together without intending to get married. | marvie11 |
| People who want children ought to get married. | marview6 |
| It is acceptible to use animals for testing medicines if it could save human lives. | meditest |
| And do you think male homosexual couples - that is gays - should be allowed to adopt a baby under the same conditions as other couples? | mgayadpt |
| All use of mobile phones while driving, including hands-free kits should be banned. | mobdban |
| Imagine an unmarried couple who split up. They have a child at primary school who remains with the mother. Do you think that the father should always be made to make maintenance payments to support the child? | mtunmar1 |
| Imagine an unmarried couple who split up. They have a child at primary school who remains with the mother. Do you think that the amount of maintenance payments that father pays for the child should depend on his income? | mtunmar2 |
| Imagine an unmarried couple who split up. They have a child at primary school who remains with the mother. Do you think that the amount of maintenance payments that father pays for the child should depend on the mother’s income? | mtunmar3 |
| People should support their country even if their country is in the wrong. | natsupp |
| Schools should teach children to obey authority. | obey |
| A person in paid work takes on an extra weekend job and is paid in cash. He does not declare it for tax and so is £500 in pocket. Do you feel this is wrong? | pay500 |
| Suppose a person wants to go on holiday but hasn’t got the money to pay for it. In your view should they save up the money beforehand rather than borrowing the money and paying it back later? | payhols |
| And now think about someone who wants to replace their broken cooker but hasn’t got the money to pay for it. In your view should they save up the money beforehand rather than borrowing the money and paying it back later? | payoven |
| Now think about someone else who wants to replace their sofa but hasn’t got the money to pay for it. In your view should they save up the money beforehand rather than borrowing the money and paying it back later? | paysofa |
| Serious complaints against the police should be investigated by an independent body, not by the police themselves. | pccompln |
| The price of a plane ticket should reflect the environmental damage that flying causes, even if this makes air travel much more expensive. | plnuppri |
| What are your views on the availability of pornographic - that is, sexually explicit - magazines and films? Do you think they should be banned altogether? | porno |
| What are your views on the availability of pornographic - that is, sexually explicit - magazines and films? Do you think they should be available in any shop for sale to adults only? | porno_alt |
| There are many ways people or organisations can protest against a government action they strongly oppose. Do you think organising public meetings to protest against the government should be allowed? | protest1 |
| Here are some more ways people or organisations can protest against a government action they strongly oppose. Do you think publishing pamphlets to protest against the government should be allowed? | protest2 |
| There are many ways people or organisations can protest against a government action they strongly oppose. Do you think organising protest marches and demonstrations should be allowed? | protest3 |
| There are many ways people or organisations can protest against a government action they strongly oppose. Do you think occupying a government office and stopping work there for several days should be allowed? | protest4 |
| There are many ways people or organisations can protest against a government action they strongly oppose. Do you think seriously damaging government buildings should be allowed? | protest5 |
| There are many ways people or organisations can protest against a government action they strongly oppose. Do you think organising a nationwide strike of all workers against the government should it be allowed? | protest6 |
| Refugees who are in danger because of their political beliefs should always be welcome in Britain. | refugees |
| Some things could be done about traffic in residential streets that are not main roads. Would be in favour having speed limits of 20 miles per hour in residential streets? | res20mph |
| Some things could be done about traffic in residential streets that are not main roads. Would be in favour having speed bumps to slow down traffic in residential streets? | resbumps |
| Some things could be done about traffic in residential streets that are not main roads. Would be in favour closing residential streets to through traffic? | resclose |
| Some people say that it is wrong to make people retire just because they have reached a certain age. Others say that older employees must retire to make way for younger age groups. What about you. Do you think it is wrong to make people retire just because they have reached a certain age? | retforce |
| There are some people whose views are considered extreme by the majority. Consider people who want to overthrow the government by revolution. Do you think such people should be allowed to hold public meetings to express their views? | revmeet |
| There are some people whose views are considered extreme by the majority. Consider people who want to overthrow the government by revolution. Do you think such people should be allowed to publish books expressing their views? | revpub |
| Do you think it is wrong if a man and a woman have sexual relations before marriage? | sexbfmar |
| There is a law in Britain against sex discrimination, that is against giving unfair preferences to men - or to women - in employment, pay and so on. Do you generally support the idea of a law for this purpose? | sexlaw |
| We would like to ask about how you think family jobs should generally be shared between men and women. Do you think it is mainly the woman who should do the household shopping? | shchore1 |
| We would like to ask about how you think family jobs should generally be shared between men and women. Do you think it is mainly the woman who should make the evening meal? | shchore2 |
| We would like to ask about how you think family jobs should generally be shared between men and women. Do you think it is mainly the woman who should do the evening dishes? | shchore3 |
| We would like to ask about how you think family jobs should generally be shared between men and women. Do you think it is mainly the woman who should do the household cleaning? | shchore4 |
| We would like to ask about how you think family jobs should generally be shared between men and women. Do you think it is mainly the woman who should do the washing and ironing? | shchore5 |
| We would like to ask about how you think family jobs should generally be shared between men and women. Do you think it is mainly the man who should repair the household equipment? | shchore6 |
| We would like to ask about how you think family jobs should generally be shared between men and women. Do you think it is mainly the man who should organise the household money and payment of bills? | shchore7 |
| What comes closest to what you think about this situation: A man gives a £5 note for goods he is buying in a corner shop. By mistake, he is given change for a £10 note. He notices, but keeps the change. Do you think it is wrong? | shopchkp |
| Thinking about a single mother with a child under school age. Do you think she has a special duty to stay at home to look after her child? | singmum1 |
| Do you think smoking should be banned in pubs and bars entirely? | smokpubs |
| Do you think smoking should be banned in restaurants entirely? | smokrest |
| Thinking about a single mother with a child of school age. Do you think she has a special duty to stay at home to look after her child? | smumsch1 |
| Social workers should put the child’s interests first, even if it means taking a child away from its natural parents. | socwchld |
| People should drive within the speed limit. | speedlim |
| A householder is having a repair job done by a local plumber. He is told that if he pays cash, he will not be charged VAT. So he pays cash. Is it wrong? | vatcheat |
| How do you feel about putting the needs of farmers before protection of wildlife? Do you think it should be encouraged? | wildlife |
| The law should always be obeyed, even if a particular law is wrong. | wronglaw |
| Do you think women should not work and stay at home after marrying and before there are children? | wwchld1 |
| Do you think women should not work and stay at home when there is a child under school age? | wwchld2 |
| Do you think women should not work and stay at home after the youngest child starts school? | wwchld3 |
| Do you think women should not work and stay at home after the children leave home? | wwchld4 |
