# Supplementary material for: Different Populations Agree on Which Moral Arguments Underlie Which Opinions
Source: Front Psychol. 2021 Mar 15;12:648405. doi: 10.3389/fpsyg.2021.648405 (PMC8005634; doi:10.3389/fpsyg.2021.648405)
Supplement: Supplementary file 7 [file Table_3.DOCX]

**Supplementary Table 3.** Matching items identified in the GSS and BSA. An asterisk (*) indicates that the issue positions were reverse coded in the UK version to match the US version.

| **General Social Survey items** | **British Social Attitudes items** |
| --- | --- |
| Do you think it should be possible for a pregnant woman to obtain a legal abortion if the woman wants it for any reason? | Do you think the law should allow an abortion in case the woman decides on her own she does not wish to have a child. |
| Do you think it should be possible for a pregnant woman to obtain a legal abortion if she is not married and does not want to marry the man? | Do you think the law should allow an abortion in case the woman is not married and does not wish to marry the man. |
| Do you think it should be possible for a pregnant woman to obtain a legal abortion if the family has a very low income and cannot afford any more children? | Do you think the law should allow an abortion in case the couple cannot afford any more children. |
| Do you think it should be possible for a pregnant woman to obtain a legal abortion if there is a strong chance of serious defect in the baby? | Do you think the law should allow an abortion in case there is a strong chance of a defect in the baby. |
| Do you think it should be possible for a pregnant woman to obtain a legal abortion if the woman’s own health is seriously endangered by the pregnancy? | Do you think the law should allow an abortion in case the woman’s health is seriously endangered by the pregnancy. |
| Do you think it should be possible for a pregnant woman to obtain a legal abortion if she became pregnant as a result of rape? | Do you think the law should allow an abortion in case the woman became pregnant as a result of rape. |
| Do you favor the death penalty for persons convicted of murder? | Are you in favour of the death penalty for murder? |
| Do you think a person has the right to end his or her own life if this person has an incurable disease? | Suppose a person has a painful incurable disease. Do you think that doctors should be allowed by law to end the patient’s life, if the patient requests it? |
| What is your opinion about a married person having sexual relations with someone other than the marriage partner–is it wrong? | What about a married person having sexual relations with someone other than his or her partner, is it always wrong? |
| Consider a man who admits that he is a homosexual. Should such a person be allowed to teach in a college or university? | Now I would like you to tell me whether, in your opinion, it is acceptable for a homosexual person to be a teacher in a college or university? |
| What about sexual relations between two adults of the same sex–do you think it is always wrong? | What about sexual relations between two adults of the same sex, is it wrong? |
| Do you think the use of marijuana should be made legal? | Smoking cannabis (marijuana) should be legalised. |
| Consider a person who advocates doing away with elections and letting the military run the country. If such a person wanted to make a speech in your community – should he be allowed to speak? | There are some people whose views are considered extreme by the majority. Consider people who want to overthrow the government by revolution. Do you think such people should be allowed to hold public meetings to express their views? |
| Consider a person who advocates doing away with elections and letting the military run the country. Suppose he wrote a book advocating doing away with elections and letting the military run the country. Somebody in your community suggests that the book be removed from the public library. Would you favor removing it? | There are some people whose views are considered extreme by the majority. Consider people who want to overthrow the government by revolution. Do you think such people should be allowed to publish books expressing their views?* |
| There’s been a lot of discussion about the way morals and attitudes about sex are changing in this country. If a man and woman have sex relations before marriage – do you think it is wrong? | Do you think it is wrong if a man and a woman have sexual relations before marriage? |
| Women should take care of running their homes and leave running the country up to men. | We would like to ask about how you think family jobs should generally be shared between men and women. Do you think it is mainly the woman who should do the household cleaning? |
| In general - would you say that people should obey the law without exception rather than there are exceptional occasions on which people should follow their consciences even if it means breaking the law? | The law should always be obeyed, even if a particular law is wrong. |
| People who want children ought to get married. | People who want children ought to get married. |
| Both the husband and the wife should contribute to the household income. | Both the man and woman should contribute to the household income. |
| Do you personally think it is wrongfor a woman to have an abortion if there is a strong chance of serious defect in the baby. | Do you personally think it is wrong for a woman to have an abortion if there is a strong chance of serious defect in the baby? |
| Do you personally think it is wrong for a woman to have an abortion if the family has a very low income and cannot afford any more children. | Do you personally think it is wrong for a woman to have an abortion if the family has a very low income and cannot afford any more children? |
| People should support their country even if the country is in the wrong. | People should support their country even if their country is in the wrong. |
| For certain problems - like environmental pollution - international bodies should have the right to enforce solutions. | For certain problems, like environment pollution, international bodies should have the right to enforce solutions. |
| America should follow its own interests - even if this leads to conflicts with other nations. | Britain should follow its own interests, even if this leads to conflicts with other nations. |
| Foreigners should not be allowed to buy land in America. | Foreigners should not be allowed to buy land in Britain. |
| American television should give preference to American films and programs. | Britain’s television should give preference to British films and programmes. |
| Ethnic minorities should be given government assistance to preserve their customs and traditions. | Ethnic minorities should be given government assistance to preserve their customs and traditions. |
